# Exploring an innovative decellularization protocol for porcine nerve grafts: a translational approach to peripheral nerve repair

**DOI:** 10.3389/fnana.2024.1380520

**Published:** 2024-03-19

**Authors:** Luisa Muratori, Alessandro Crosio, Giulia Ronchi, Debora Molinaro, Pierluigi Tos, Arianna B. Lovati, Stefania Raimondo

**Affiliations:** ^1^Department of Clinical and Biological Sciences, Neuroscience Institute Cavalieri Ottolenghi (NICO), University of Turin, Turin, Italy; ^2^UOC Traumatology-Reconstructive Microsurgery, Department of Orthopedics and Traumatology, CTO Hospital, Turin, Italy; ^3^Reconstructive Microsurgery and Hand Surgery Unit, ASST Pini-CTO, Milan, Italy; ^4^Cell and Tissue Engineering Laboratory, IRCCS Istituto Ortopedico Galeazzi, Milan, Italy

**Keywords:** peripheral nerve injuries, orthopedic trauma, pig, nerve repair, decellularized nerve

## Abstract

**Introduction:**

Peripheral nerves are frequently affected by lesions caused by traumatic or iatrogenic damages, resulting in loss of motor and sensory function, crucial in orthopedic outcomes and with a significant impact on patients’ quality of life. Many strategies have been proposed over years to repair nerve injuries with substance loss, to achieve musculoskeletal reinnervation and functional recovery. Allograft have been tested as an alternative to the gold standard, the autograft technique, but nerves from donors frequently cause immunogenic response. For this reason, several studies are focusing to find the best way to decellularize nerves preserving either the extracellular matrix, either the basal lamina, as the key elements used by Schwann cells and axons during the regenerative process.

**Methods:**

This study focuses on a novel decellularization protocol for porcine nerves, aimed at reducing immunogenicity while preserving essential elements like the extracellular matrix and basal lamina, vital for nerve regeneration. To investigate the efficacy of the decellularization protocol to remove immunogenic cellular components of the nerve tissue and to preserve the basal lamina and extracellular matrix, morphological analysis was performed through Masson’s Trichrome staining, immunofluorescence, high resolution light microscopy and transmission electron microscopy. Decellularized porcine nerve graft were then employed in vivo to repair a rat median nerve lesion. Morphological analysis was also used to study the ability of the porcine decellularized graft to support the nerve regeneration.

**Results and Discussion:**

The decellularization method was effective in preparing porcine superficial peroneal nerves for grafting as evidenced by the removal of immunogenic components and preservation of the ECM. Morphological analysis demonstrated that four weeks after injury, regenerating fibers colonized the graft suggesting a promising use to repair severe nerve lesions. The idea of using a porcine nerve graft arises from a translational perspective. This approach offers a promising direction in the orthopedic field for nerve repair, especially in severe cases where conventional methods are limited.

## 1 Introduction

Peripheral nerve injuries result in partial or total loss of function with significant consequences in orthopedic outcomes and impairment in quality of life of affected patients (Lavorato et al., 2023). Even if peripheral nerves retain an intrinsic capability to regenerate spontaneously, this natural process is often unsatisfactory particularly in cases of severe injuries involving significant tissue loss. Therefore, achieving musculoskeletal reinnervation and improving the functional recovery are important medical challenges, with fully effective treatments yet to be established (Thibodeau et al., 2022). To date, the gold standard surgical procedure for nerve injuries with substance loss is the autograft technique. However, this approach has significant drawbacks, including limited graft availability, the necessity of a two-stage surgical process, and potential adverse effects that can lead to morbidity at the donor site (Hoben et al., 2018). In order to improve the outcome of such injuries, many attempts have been made to develop devices that can be used to bridge nerve gap and support nerve regeneration (Fornasari et al., 2020; Wu et al., 2023). Among these, an increasing number of strategies proposes the implantation of “decellularized nerve allografts” characterized by the removal of cellular antigens while maintaining the structure of a native nerve made of extracellular matrix (ECM), basal lamina, and endoneurial tubes. These components promote the regenerative process allowing Schwann cell (SC) proliferation, migration, and axonal elongation (Contreras et al., 2022, 2023). Furthermore, ECM plays a role as a supportive structure through chemical interaction among SCs by soluble signaling, receptor pathways, and mechanotransduction (Armstrong et al., 2007; Urbanski et al., 2016; Prest et al., 2018; Xu et al., 2020). These evidences highlight the importance of ECM maintenance during nerve regeneration. Over time, numerous protocols have been developed that utilize a mix of chemical and biological agents along with physical methods to create effective decellularized nerve grafts, which can facilitate and ensure nerve regeneration (Sondell et al., 1998; Armstrong et al., 2007; El Soury et al., 2021).

In this study, a novel decellularization protocol currently used to decellularize tendons (Bottagisio et al., 2019) was applied on porcine superficial peroneal nerves to obtain decellularized nerve grafts. The first aim of the present study was to assess the effectiveness of the decellularization procedure applied for the first time on superficial peroneal pig nerves. The secondary goal was to conduct *in vivo* experiments using these decellularized porcine nerve grafts to repair median nerve injuries in rats, to determine the potential of this xenograft model in supporting nerve regeneration.

## 2 Materials and methods

### 2.1 Study design

**Figure 1:**
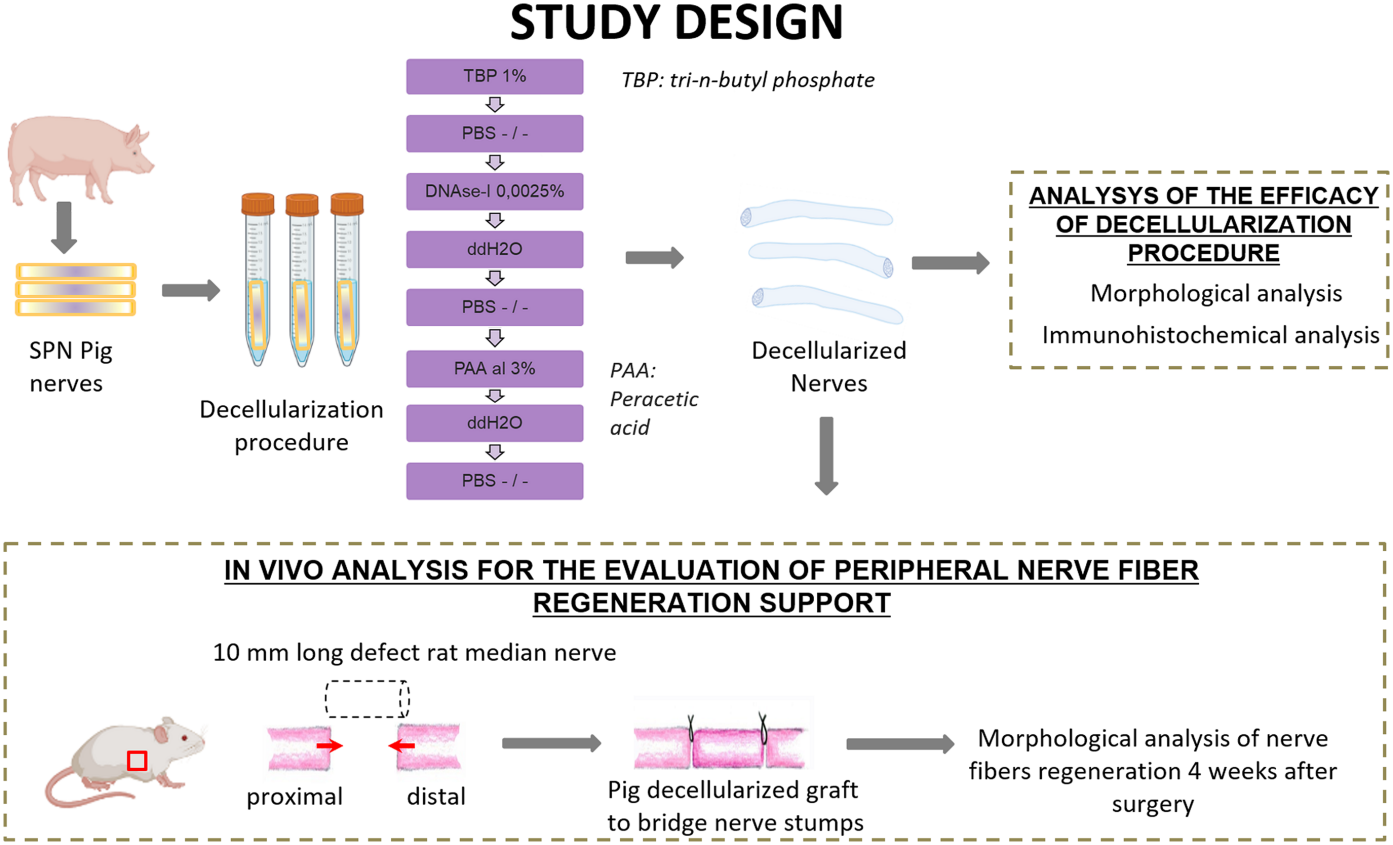
Schematic representation of experimental design and analysis. Created with BioRender.com.

Four superficial peroneal nerves (SPN) were harvested from two 6-months old pigs as waste anatomical tissues from subjects euthanized for an unrelated study (Authorization #771/2020-PR, ID 185D1.6.EXT.0). Each nerve was cut to create nerve segments of 1 cm length and a total of 18 nerve segments were obtained. Nine nerve segments were used as controls, and nine segments underwent the decellularization procedure. Once obtained decellularized grafts, they were divided into two study groups, one for the analysis of the efficacy of the decellularization procedure and the second for the application of the porcine decellularized nerve graft *in vivo* to repair median nerve injuries in rats (Figure 1).

### 2.2 Decellularization protocol

The decellularization of the nerve segments followed a method outlined for tendons in a previous study (Bottagisio et al., 2019). In summary, nerves were treated with 1% tri-n-butyl phosphate (TBP) in a Tris–HCl buffer, followed by rinsing and storage steps to eliminate detergents. DNAse-I and 3% peracetic acid (PAA) treatments were then applied to further process the samples (Figure 1). Subsequent rinses in distilled water and PBS were conducted before their use. Specifically, some samples were fixed in 4% paraformaldehyde for histological analyses, and 2.5% glutaraldehyde for TEM. Samples dedicated to the *in vivo* study were stored at −80°C until use.

### 2.3 Morphological evaluation of decellularized porcine nerves

To evaluate the effectiveness of the decellularization protocol applied on porcine superficial peroneal nerves, morphological analysis has been carried out comprising Masson’s Trichrome staining, immunofluorescence, high resolution light microscopy and Transmission Electron Microscopy (TEM).

#### 2.3.1 Paraffin embedding

Nerve samples were fixed by immediate immersion in 4% paraformaldehyde for 2 h, washed in a solution of 0.2% glycine in 0.1 M phosphate buffer (pH 7.2), and embedded in paraffin. Specimens were cut 10 μm thick using a Leica RM2125 microtome and processed for histological and immunofluorescence analysis.

#### 2.3.2 Masson’s trichrome staining

For Masson’s trichrome staining, a Masson trichrome with aniline blue kit (Bio-Optica) was used. This method is a three-color staining that allows to identify collagen (blue), cytoplasm (red), and nuclei (black). As a first step, a mixture of Weigert’s iron hematoxylin were combined together to stain slides. After an incubation with alcoholic picric acid and a wash in distilled water, sections were stained with Ponceau acid fuchsin, and lastly with aniline blue for collagen detection. As a final step, sections were rapidly dehydrated in ethanol, cleared in xylol/Bioclear (Bio-Optica) and mounted in DPX (Fluka).

#### 2.3.3 Immunofluorescence and confocal microscopy

Sections were rinsed in PBS, blocked with normal goat serum (1% in PBS–Triton 0.1%) for 1 h and incubated overnight with the primary antibody. After primary antibody(ies) incubation, sections were washed three times in PBS and incubated for 1 h in a solution containing the secondary antibodies: Alexa 488 anti-Mouse, Cy3 anti-Rabbit (Life Technologies) in order to recognize the species of primary antibodies. After three washes in PBS, sections were mounted with a Dako fluorescence mounting medium and stored at 4°C before being analyzed. The sections were incubated in a solution containing the following primary antibodies: anti-PAN-NF (monoclonal, mouse, Sigma Aldrich), anti S-100 (polyclonal, rabbit, Sigma Aldrich), anti-peripherin (polyclonal rabbit, Sigma Aldrich). Nuclei were stained with 4,6-diamidino-2-phenylindole (DAPI, Sigma) diluted 1:1,000 in PBS. Finally, sections were mounted with a Dako fluorescence mounting medium. Images were acquired using a Zeiss LSM800 confocal laser microscopy system (Zeiss, Jena, Germany).

#### 2.3.4 Resin embedding, high resolution light microscopy, and transmission electron microscopy

Native and decellularized superficial peroneal nerves were fixed by immediate immersion in 2.5% glutaraldehyde (*SIC*, Società Italiana Chimici) in 0.1 M phosphate buffer (pH 7.4) for 5–6 h, at 4°C. Samples were then post-fixed in 2% osmium tetroxide (*SIC*, Società Italiana Chimici) for 2 h and dehydrated in passages in ethanol (Sigma Aldrich) from 30 to 100% (5 min of each passage). After two 7 min passages in propylene oxide and overnight in a 1:1 mixture of propylene oxide (Sigma Aldrich) and Glauerts’ mixture of resins, the samples were embedded in Glauerts’ mixture of resins (made of equal parts of Araldite M and Araldite Harter, HY 964, Sigma Aldrich). Into the resin mixture, 0.5% of the plasticizer dibutyl phthalate (Sigma Aldrich) was added. For the final step, 2% of accelerator 964 was added to the resin in order to promote the polymerization of the embedding mixture, at 60°C. Semi-thin sections (2.5 μm thick) were cut using an Ultracut UCT ultramicrotome (Leica Microsystems, Wetzlar, Germany) and stained with 1% toluidine blue and 2% borate in distilled water for high resolution light microscopy. A DM4000B microscope equipped with a DFC320 digital camera was used for high resolution light microscopy.

For transmission electron microscopy analysis (TEM), ultrathin (70 nm thick) sections were obtained with the same ultramicrotome, stained with a solution of 4% UAR-EMS uranyl acetate replacement in distilled water, and analyzed using a JEM-1010 transmission electron microscope (JEOL, Tokyo, Japan) equipped with a Megaview-III digital camera and a Soft-Imaging-System (SIS, Münster, Germany) for the computerized acquisition of the images.

### 2.4 *In vivo* study

To study the ability of porcine decellularized nerves graft to support peripheral nerve regeneration, decellularized nerves graft were implanted and used to repair median nerve injuries with substance loss in rats.

#### 2.4.1 Ethic statement

A total of 3 adult Wistar rats weighing approximately 230–250 g (Charles River Laboratories, Milan, Italy) were used for this study. Animals were housed in large cages at the animal facility of Neuroscience Institute Cavalieri Ottolenghi (NICO; Ministerial authorization DM 1822010-A 3–11-2010) in a humidity and temperature-controlled room with 12 h light/dark cycles. Free access to water and standard chow was provided. Human endpoint criteria were adopted to adequately measure and minimize any animal pain, discomfort, or distress. The study conditions conformed to the guidelines of the European Union’s Directive EU/2010/63. In addition, approval by the Ethic Experimental Committee of the University of Turin (Ministry of Health project number 692/2020) was obtained before the research began.

#### 2.4.2 Surgical procedure

**Figure 2:**
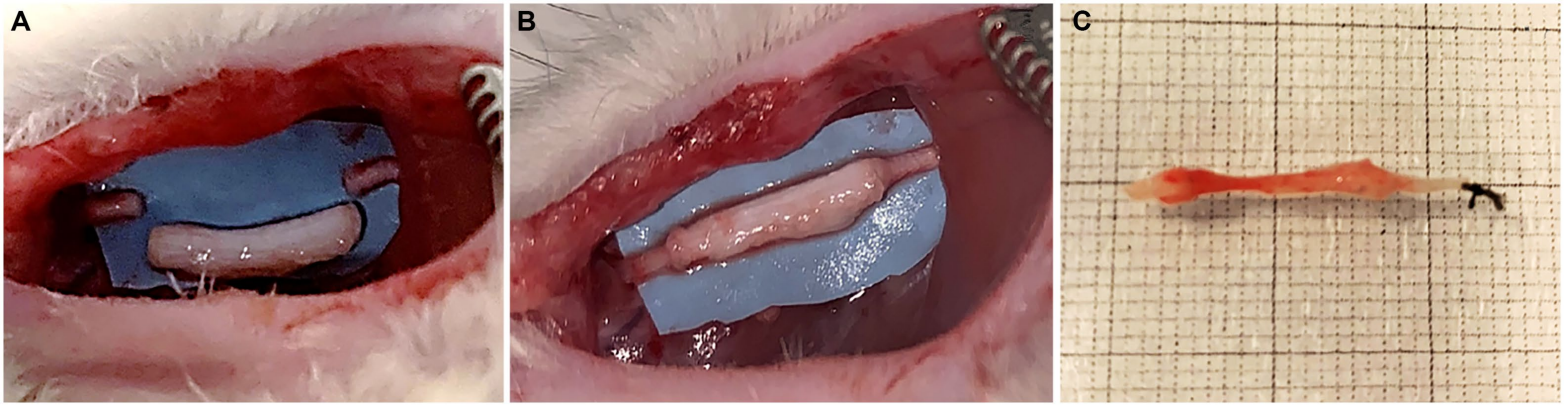
Surgical procedure: median nerve resection (10 mm gap) **(A)**; repair with porcine decellularized superficial peroneal nerve graft **(B)**; regenerated nerve harvested 4 weeks after implantation **(C)**.

Surgeries were performed under general anesthesia, with ketamine hydrochloride (2.5 mg/kg) and xylazine (50 mg/kg) by intraperitoneal injection. All surgical procedures were carried out under a surgical microscope with a magnification range from 3,6x to 22,3x used by the surgeon according to the surgical steps. Nerve lesion was performed on median nerves. The median nerve of both forelimbs was severed and a nerve segment of 8 mm was removed and repaired with 1 cm decellularized superficial peroneal nerve. The nerve graft was sutured with 9/0 epineurial stitch at each end (Figures 2A,B). After 4 weeks, rats were euthanized through anesthetic overdose of ketamine and xylazine by intraperitoneal injection. The surgical site was exposed, regenerated nerve samples were harvested and processed for morphological evaluation of nerve regeneration.

#### 2.4.3 Resin embedding, high resolution light microscopy and TEM

Regenerated nerves were processed for resin embedding, high resolution light microscopy and TEM according to the aforementioned procedures (see section 2.3.4). Particularly for the *in vivo* study, in order to understand if regenerated fibers reached the end of the graft, only the distal portions of the sample were cut and analyzed by high resolution light microscopy and TEM.

#### 2.4.4 Histological procedures

Samples were processed for paraffin embedding as previously described (see section 2.3.1), transversal cross sections (10 μm thickness) of the whole nerve were obtained starting from the distal portion until the proximal, in order to evaluate the course of the regenerated fibers on the whole sample.

#### 2.4.5 Immunofluorescence

In order to identify the presence of regenerated nerve fibers and ECM components, immunofluorescence analysis was performed on the proximal, middle and distal portions of regenerated nerves. For this purpose, sections were dewaxed, rinsed in PBS, blocked with normal goat serum (1% in PBS–Triton 0.1%) for 1 h and incubated overnight with primary antibodies. After primary incubation, the sections were washed three times in PBS and incubated for 1 h in a solution containing the secondary antibodies: Alexa 488 anti-Mouse, Cy3 anti-Rabbit (Life Technologies) in order to recognize the species of primary antibodies. After three washes in PBS, sections were mounted with a Dako fluorescence mounting medium and stored at 4°C before being analyzed. The sections were incubated in a solution containing the following primary antibodies: anti-PAN-NF (monoclonal, mouse, Sigma Aldrich), anti S-100 (polyclonal, rabbit, Sigma Aldrich), anti-laminin (polyclonal rabbit, Sigma Aldrich). Nuclei were stained with 4,6-diamidino-2-phenylindole (DAPI, Sigma) diluted 1:1,000 in PBS. Finally, sections were mounted with a Dako fluorescence mounting medium. Images were acquired using a Zeiss LSM800 confocal laser microscopy system (Zeiss, Jena, Germany).

## 3 Results

The first aim of the present study was to test the efficacy of the decellularization method currently used to decellularize horse tendons in order to evaluate its possible application as a nerve graft. The procedure effectiveness was assessed by determining if immunogenic nuclear and cellular components of the nerve tissue were removed, and whether the ECM was preserved.

The second aim was to evaluate the ability of the porcine decellularized nerve graft to support nerve regeneration after injury using it to repair median nerve lesions in rats.

### 3.1 Evaluation of overall nerve morphology and connective tissue structure after decellularization protocol

In order to evaluate the overall morphology and connective tissue statement of porcine decellularized superficial peroneal nerve, Masson’s Trichrome staining was performed on decellularized and native nerves.

**Figure 3:**
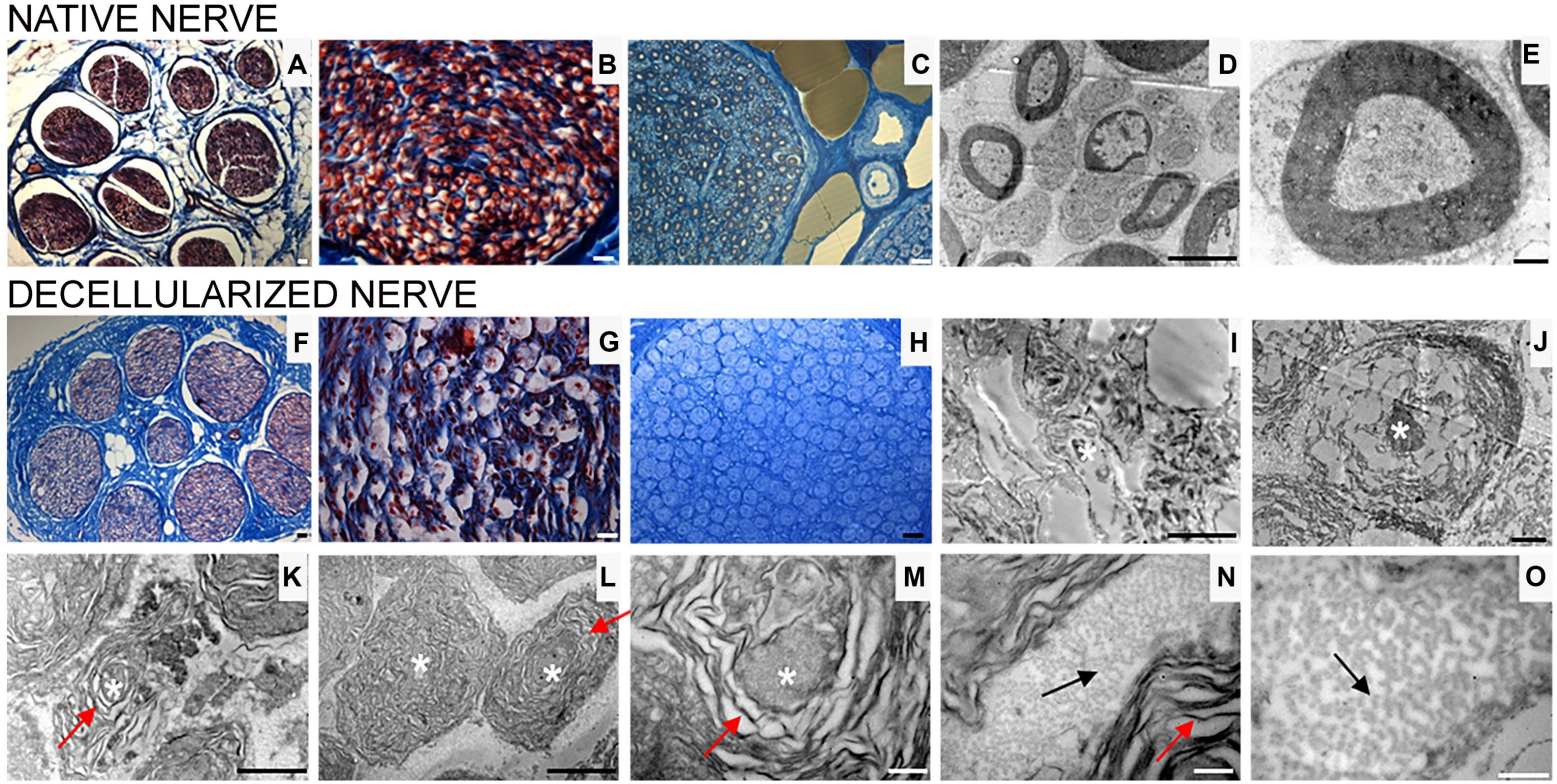
Masson’s trichrome staining **(A,B,F,G)**, high resolution light microscopy **(C,H)** and transmission electron microscopy **(D,E,I–O)** performed on native **(A–E)** and decellularized **(F–O)** porcine superficial peroneal nerves. Scale bar **(A–C,F–H)** = 20 μm; **(D,I)** = 10 μm; **(E,J)** = 0,2 μm; **(K)** = 2 μm, **(L)** = 1 μm; **(M–O)** = 0.5 μm. Asterisk marks degenerating axons; red arrows mark enlarged lamellae of Schwann cells; black arrows mark collagen fibrils.

Transversal cross sections of superficial peroneal nerves displayed the typical histological organization of a healthy nerve characterized by many nerve fascicles, each surrounded by perineurium, and adipose tissue between fascicles, held together by epineurium, the outermost layer of connective tissue (Figures 3A,B).

High resolution light microscopy on semi- thin cross sections stained with Toluidine Blue, highlights the morphology of nerve fibers within nerve fascicles. Moreover, adipose tissue is well detectable (Figure 3C). Ultrastructural analysis, performed using TEM, allows to identify the presence of unmyelinated and myelinated fibers (Figure 3D); at higher magnifications, an axon surrounded by a Schwann cell to form myelin sheath with lamellae spirally arranged is detectable (Figure 3E).

In decellularized nerves, Masson’s Trichrome staining allowed to verify that the outer epineurium was still present as well as the perineurium that surrounded each fascicle (Figure 3F). The amount of adipose tissue decreased. At higher magnifications (Figure 3G), nerve fibers were barely detectable compared with native nerves.

To further evaluate the effectiveness of the decellularization protocol on porcine superficial peroneal nerves, high resolution light microscopy and TEM analyses were performed (Figures 3H–O): native nerve displayed axons surrounded by myelin sheath, adipose tissue and blood vessels (Figure 3C), in decellularized nerve myelin sheath is not clearly identifiable, however the presence of connective tissue around nerve fascicles was still present (Figure 3H) Ultrastructural analysis displayed empty space and scattered myelin around degenerating axons (Figures 3I,J, asterisks), collapsed axons with enlarged disintegrated myelin sheath were detectable (Figures 3K–M, red arrows). At the same time, a well-preserved collagen made of collagen fibrils was clearly visible (Figures 3N,O, black arrows).

### 3.2 Evaluation of axonal and glial components after the decellularization protocol

In order to detect immunogenic components such as axonal filaments, nuclei and myelin, specific axonal, glial and nuclear markers were tested in native and decellularized porcine nerves.

**Figure 4:**
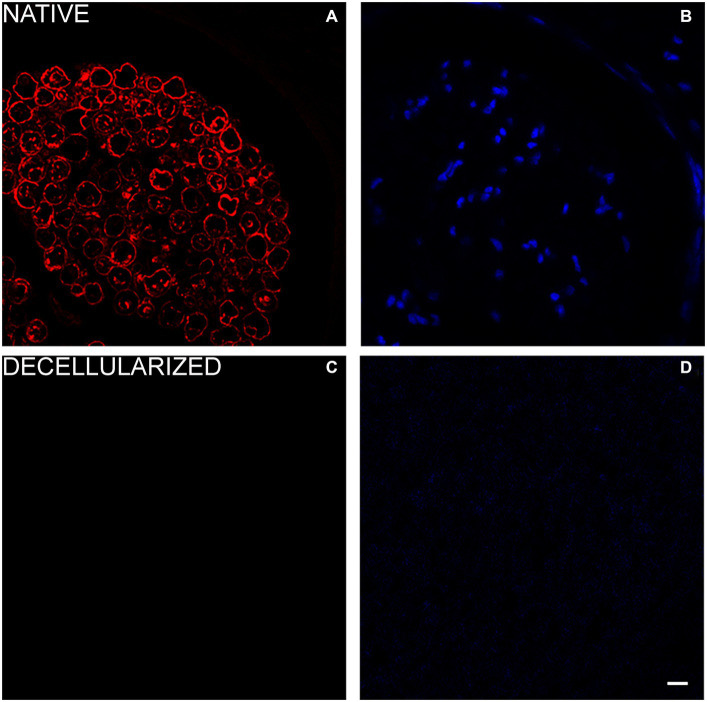
Immunofluorescence analysis to detect glial cells (S100 in red—**A,C**) and nuclei (DAPI in blue—**B,D**) in native **(A,B)** and decellularized porcine nerves **(C,D)**. Scale bar = 20 μm.

For glial components an immunostaining with S100 was performed and DAPI as nuclear staining. Native porcine nerves showed the normal morphology in which Schwann cells form myelin sheath around axons; many nuclei were clearly visible in blue (DAPI) (Figures 4A,B). In decellularized nerves, no immunostaining was found for S100 and DAPI (Figures 4C,D).

**Figure 5:**
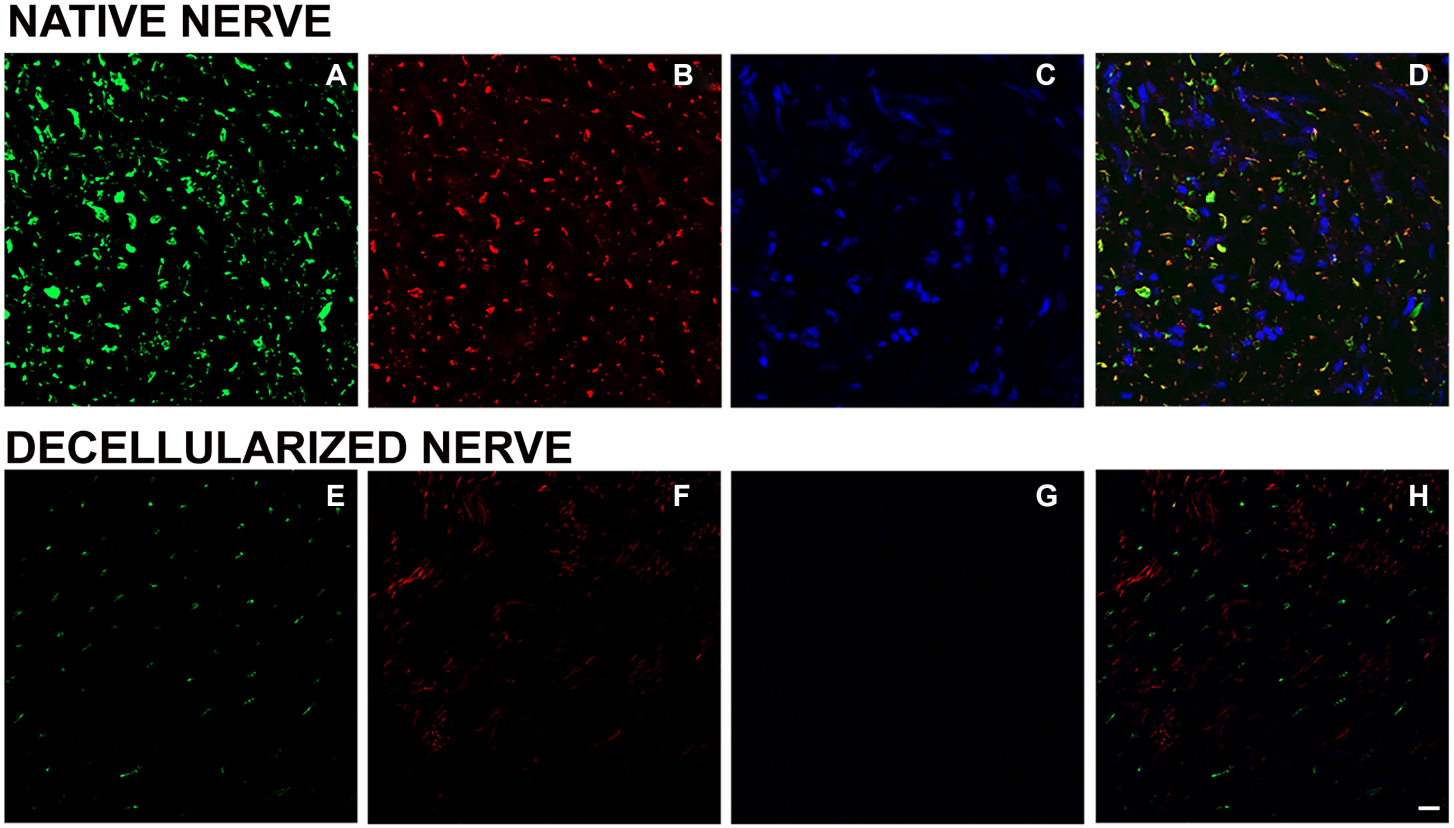
Immunofluorescence analysis to detect axonal components and nuclei in native **(A–D)** and decellularized nerves **(E–H)**. Green: neurofilament **(A,E)**; red: peripherin **(B,F)**; DAPI: blue **(C,G)**; merging **(D,H)**. Scale bar = 20 μm.

To investigate the presence of axonal components, anti-neurofilament -a typical protein expressed by axons - and peripherin -a type III intermediate protein expressed by unmyelinated fibers - were used in a double immunostaining either on native (Figures 5A–D) and decellularized nerves (Figures 5E–H). Results revealed the presence of a few axons immunopositive for peripherin (red) and neurofilament (green) in decellularized nerve demonstrating a low presence of this axonal proteins. In native nerves, the same staining showed the presence of axonal neurofilament and peripherin according to the expected morphology.

### 3.3 Macroscopic assessment of implantation site

Four weeks after surgery, surgical site was assessed for each rat that have been undergone the implantation of decellularized porcine nerve graft, in order to evaluate the possible presence of inflammatory reaction or neuroma formation. No rats showed signs of inflammation or neuroma formation (Figure 2C), moreover no rats showed distress throughout the postoperative time.

### 3.4 Morphological assessment of nerve regeneration along the decellularized nerve graft

Rat median nerves repaired with decellularized porcine superficial peroneal nerve graft were harvested 4 weeks after implantation to investigate the ability of the decellularized graft to support nerve regeneration.

In order to study the morphological pathway of regenerated fibers, three different portions of the regenerated graft were analyzed: proximal, middle and distal.

**Figure 6:**
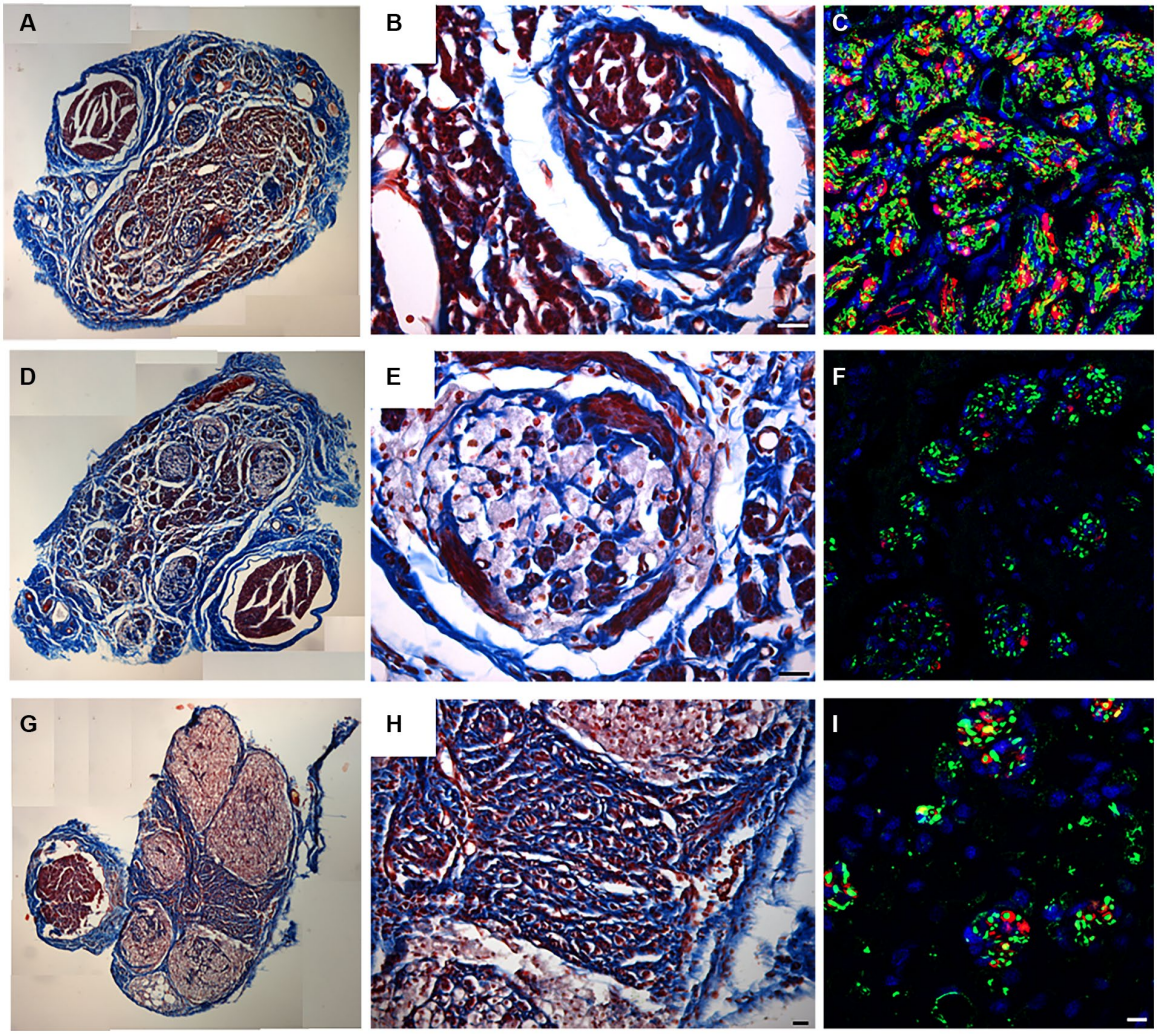
Masson’s trichrome staining **(A,B,D,E,G,H)** and IHC for neurofilament (Green) and S100 (red), DAPI in Blue **(C,F,I)** performed to identify the presence of regenerated fibers on proximal **(A–C)**, middle **(D–F)** and distal **(G–I)** level. Scale bar = 20 μm.

Masson’s trichrome staining was employed to assess the overall structure of the regenerated nerve at different portions, showing the morphology of the connective tissue surrounding nerve fascicles and the presence of red staining within the regenerated nerve (Figures 6A–G).

In the proximal portion (Figure 6A), decellularized fascicles were not detectable compared with the other portions (Figures 6D,G). At higher magnifications, regenerated fascicles were clearly visible together with a large regenerated red zone detectable by Masson’s Trichrome staining (Figure 6B).

In order to identify the presence of newly regenerated nerve fibers, immunofluorescence analysis using axonal (Neurofilament) and glial markers (S100) allowed to clearly detect axons and Schwann cells at the proximal portion (Figure 6C). Results clearly showed the typical morphology of a regenerated nerve fully colonized by new nerve fibers immunopositive for S100 and Neurofilament (Figure 6C).

Shifting toward the center of the regenerated graft, decellularized fascicles became visible (Figure 6D) and colonized by regenerated nerve fibers identifiable either within porcine fascicles or among them (Figure 6E). Immunostaining confirmed the presence of regenerated fibers (Figure 6F).

Considering the distal portion (Figures 6G–I), the presence of large nerve fascicles was identified, corresponding to the decellularized porcine nerve, characterized by weak red staining (Figure 6G). Interestingly among these, many regenerated axons were detectable by an intense red staining and surrounded by connective tissue (Figure 6H). The immunofluorescence analysis reported in Figure 6I demonstrated that regenerated fibers, stained with neurofilaments and glial markers, reached the distal portion of the graft.

### 3.5 Ultrastructural analysis of regenerated nerve

**Figure 7:**
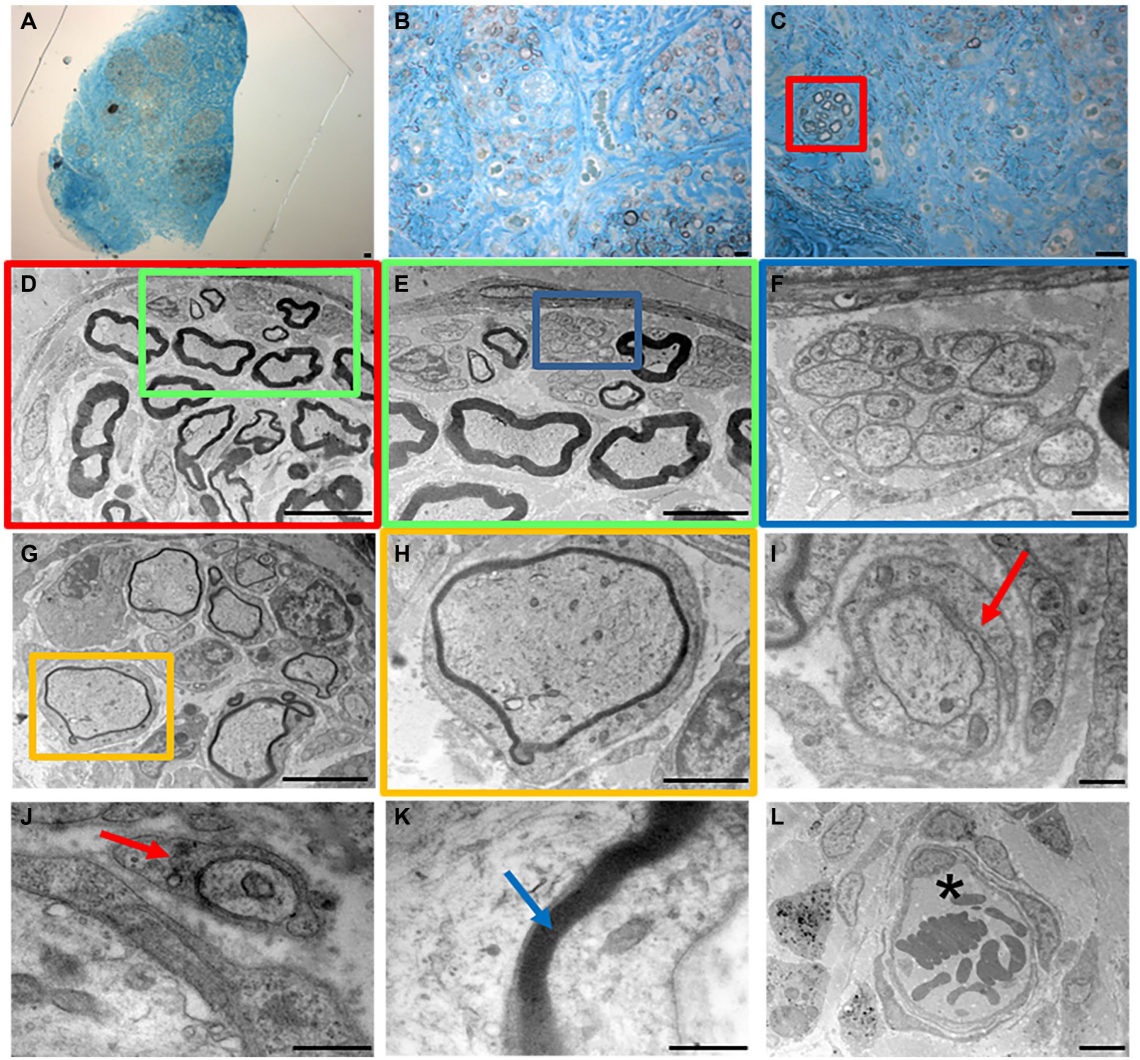
High resolution light microscopy **(A–C)** and transmission electron microscopy **(D–L)** at distal portion of the regenerated nerve 4 weeks after surgery. Scale bar: **(A–C)** = 20 μm; **(D)** = 10 μm; **(E–G)** = 5 μm; **(F)** = 1 μm; **(H)** = 2 μm; **(I–L)** = 0.5 μm. Red arrows mark enveloping Schwann cells; blue arrow marks myelin; asterisk marks a blood vessel.

High resolution light microscopy on transversal cross-section of rat median nerve repaired with decellularized superficial peroneal nerve graft revealed the presence of cells, nuclei and blood vessels at distal portions 4 weeks after implant (Figures 7A–C). Interestingly, a well-defined bundle of nerve fibers surrounded by connective tissue was clearly evident within the regenerated nerve (Figure 7C).

To further improve the morphological evaluation of the regenerated nerve, TEM analysis enabled the study of its ultrastructural features characterized by both myelinated and unmyelinated fibers (Figures 7D–F).

Myelinated fibers were also randomly detectable within the nerve (Figures 7G,H), and an enveloping Schwann cell during the early phase of axonal myelination (Figures 7I,J, red arrows). Finally, ultrastructural features of packed lamellae made of Schwann cells membrane were easily detectable (Figure 7K, blue arrow) together with blood vessels with erythrocytes (Figure 7L, asterisk).

### 3.6 Evaluation of extracellular matrix preservation

Because ECM (extracellular matrix) molecules play a crucial role in creating a specific and supportive physical and chemical environment for cell survival, as well as for the proliferation and migration of Schwann Cells during nerve regeneration, the study focused on preserved laminin (Armstrong et al., 2007; de Luca et al., 2014). Indeed, laminin is a significant substrate not only for Schwann cell functions but also for the axonal elongation.

**Figure 8:**
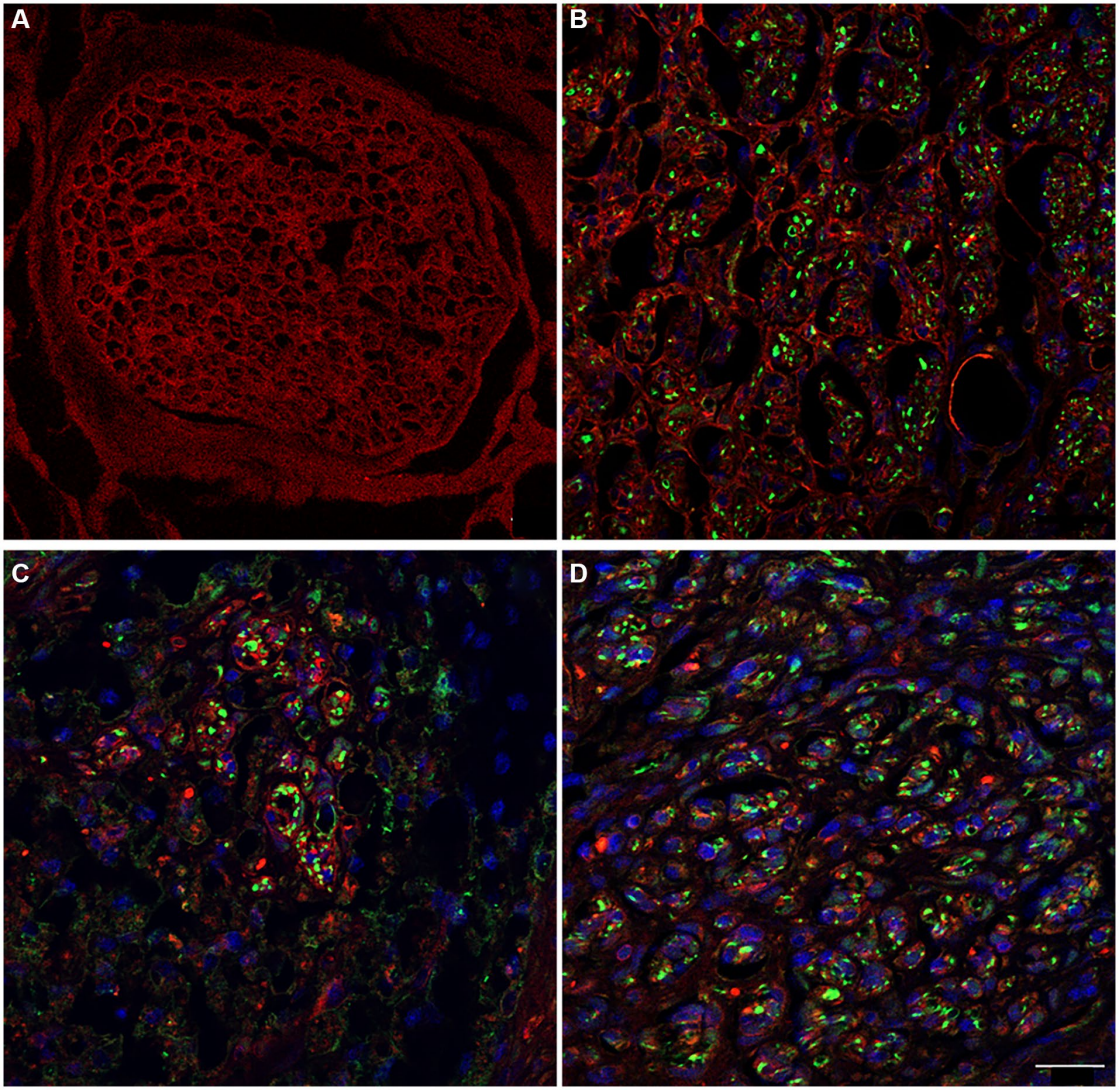
The immunoreactivity for laminin in decellularized porcine nerve **(A)** and in proximal **(B)**, middle **(C)**, distal **(D)** portion of the regenerated nerve displaying the preservation of the ECM (red) colonized by regenerating axons (green) and cellular nuclei (DAPI). Scale bar = 20 μm.

Immunolabeling for anti-laminin in the decellularized porcine superficial peroneal nerve indicated the preservation of this crucial ECM component post-decellularization (Figure 8A). Similarly, in the rat median nerve repaired with the decellularized graft, a comparable immunopositivity was observed. This was evident in the proximal (Figure 8B), middle (Figure 8C), and distal (Figure 8D) portions of the regenerated nerve, where many neurofilament-positive axons (green) were seen within endoneurial tubes that also showed positive immunoreactivity for laminin.

## 4 Discussion

In the field of tissue engineering, the development of decellularized nerve grafts is a topic of ongoing research. These grafts are being explored as potential alternatives to the traditional autograft technique, especially for repairing nerve injuries with longer gaps (over 3 cm). Studies involve assessing the regenerative potential of various decellularization protocols on animal models to understand their effectiveness in nerve regeneration. This research is crucial for advancing the treatment of severe nerve injuries where traditional methods may not be sufficient (Garcia-Garcia et al., 2023). Decellularized nerve grafts should provide a suitable substrate for nerve regeneration in which the conservation of ECM components plays a crucial role for peripheral nerve regeneration and axonal guidance (Hsu et al., 2023; Mahdian et al., 2023). The currently available decellularized methods are time and effort consuming (El Soury et al., 2021). Consequently, there is an ongoing effort to develop and propose new decellularization procedures and strategies. In this study, a novel decellularization method already used to obtain decellularized tendons (Bottagisio et al., 2019) was applied for the first time on porcine nerves in order to study its effectiveness to create a valid decellularized nerve graft able to support nerve regeneration in case of injury with substance loss in rats. Since nerves and tendons share common features, such as scarcely tissue permeability (El Soury et al., 2021), it was hypothesized that the use of this decellularization protocol in nerves could efficiently produce biocompatible and well-structured decellularized nerve grafts. The study successfully demonstrated that the decellularization method used for horse tendons can be effective in preparing porcine superficial peroneal nerves for grafting. This indicates a potential application in nerve grafts, as evidenced by the removal of immunogenic components and preservation of the ECM. Detailed morphological assessments through various staining and microscopy techniques revealed significant insights into the structural integrity and changes post-decellularization. The preservation of key nerve structures alongside some level of degradation points to areas for further optimization. Morphological analysis performed on decellularized porcine nerves showed that glial components have been successfully removed together with nuclei. The absence of immunogenic and glial markers in decellularized nerves indicates successful decellularization. This is a crucial factor in preventing host immune response post-transplantation. Masson’s trichrome staining highlighted the preservation of the overall connective tissue structure made of epineurium that surround the whole nerve, perineurium surrounding nerve fascicles and endoneurium surrounding individual nerve fibers. Ultrastructural analysis performed by TEM confirmed the conservation of collagen fibrils within the decellularized graft.

The second aim of this study was to evaluate the ability of the decellularized nerve graft to support nerve regeneration *in vivo* using a xenograft model in which a decellularized porcine nerve was used to repair a median nerve injury in rats. The use of decellularized porcine nerve grafts in rat median nerve injury models showed promising results. Despite the limitation of this study is related to the number of rats employed, it represents an initial proof of concept of the efficacy of decellularized xenografts in nerve repair. Indeed, the study observed nerve regeneration, suggesting that these grafts can effectively support nerve repair processes. Masson’s Trichrome staining allowed to identify the organization of regenerated nerve fibers and connective tissue conditions in regenerated nerve showing a substantial presence of nerve fibers starting from the proximal toward the distal portion of the nerve. In order to demonstrate the presence of such regenerated fibers, immunofluorescence analysis allowed the detection of axons, SC forming myelin and nuclei at the proximal, middle and distal portions. Particularly, according to the regenerative time (4 weeks of follow-up), TEM revealed the presence of many unmyelinated fibers, enveloping SC forming myelin sheath and myelinated fibers at the distal portion. Finally, the preservation of laminin in porcine nerve graft after decellularization and in regenerated median nerve of rats strengthened the specificity of the protocol to preserve ECM components that play a key role for SC proliferation, migration and axonal elongation (Armstrong et al., 2007). The preservation of ECM components like laminin, essential for nerve regeneration, underscores the potential of this decellularized graft in clinical applications. This is further supported by the successful colonization and regeneration of nerve fibers in the graft. Taken together the results achieved in this study demonstrated either the effectiveness of the decellularization protocol in removing the native nerve components, or its supporting role as nerve graft to promote axonal elongation and fiber regeneration.

The translational value of this study lies in the use of a xenograft model, a technique that involves transplanting tissue from one species to another. This approach holds significant promise in the field of regenerative medicine for replacing damaged tissues, highlighting its potential impact and utility. A previous study (Hopf et al., 2022) successfully investigated a new method to decellularize large-diameter porcine nerves combining a mild chemical disruption of cellular components with enzymatic degradation: a large acellular nerve scaffold was obtained with a preserved ECM structure, supporting the possible application of porcine nerve graft in the clinical context of nerve repair.

Indeed, actually, many porcine tissues are efficiently transplanted in human, i.e., porcine dermis used as a graft to support the repair of large skin loss after burned condition (Haller et al., 2021), or decellularized porcine heart valve scaffolds successfully implanted for heart valve tissue engineering (Leyh et al., 2003).

Starting from these evidences, the potential application of a decellularized porcine nerve graft as a xenograft model to repair human nerve injury could be suitable since porcine nerves are easy to obtain and compatible with humans considering diameter and anatomical size (Zilic et al., 2015; Wei et al., 2022).

In conclusion, the study presents significant advancements in the field of nerve repair, especially regarding the use of decellularized grafts. While promising, these findings also highlight the need for continued research to refine these methods for clinical applications. The study sets a foundation for future investigations into optimizing decellularization protocols and assessing long-term outcomes in nerve regeneration.

## Data availability statement

The raw data supporting the conclusions of this article will be made available by the authors, without undue reservation.

## Ethics statement

The animal study was approved by the Ethic Experimental Committee of the University of Turin (Ministry of Health project number 692/2020). The study conditions conformed to the guidelines of the European Union’s Directive EU/2010/63. The study was conducted in accordance with the local legislation and institutional requirements.

## Author contributions

LM: Data curation, Writing – original draft, Writing – review & editing, Investigation, Methodology. AC: Methodology, Writing – review & editing. GR: Methodology, Writing – review & editing. DM: Methodology, Writing – review & editing. PT: Writing – review & editing, Conceptualization, Supervision. AL: Conceptualization, Writing – review & editing, Methodology. SR: Writing – review & editing, Data curation, Supervision, Writing – original draft.
